# Fluorescent protein-based detection of unconjugated bilirubin in newborn serum

**DOI:** 10.1038/srep28489

**Published:** 2016-06-21

**Authors:** Sota Iwatani, Hajime Nakamura, Daisuke Kurokawa, Keiji Yamana, Kosuke Nishida, Sachiyo Fukushima, Tsubasa Koda, Noriyuki Nishimura, Hisahide Nishio, Kazumoto Iijima, Atsushi Miyawaki, Ichiro Morioka

**Affiliations:** 1 Department of Pediatrics, Kobe University Graduate School of Medicine, Kobe 6500017, Japan; 2 Department of Epidemiology, Kobe University Graduate School of Medicine, Kobe 6500017, Japan; 3 Brain Science Institute, RIKEN, Wako 3510198, Japan

## Abstract

Increased serum levels of unconjugated bilirubin are associated with the development of brain damage in newborns. In current clinical settings, there are no methods for directly determining serum levels of unconjugated bilirubin. UnaG, a fluorescent protein from Japanese eel muscle that specifically binds to unconjugated bilirubin was used in this study. Linear regression analysis was carried out to compare unconjugated bilirubin levels measured by UnaG and conventional bilirubin oxidase methods. Unconjugated bilirubin levels in the serum of newborns who were untreated or treated with phototherapy were compared. Effects of interfering factors in the serum (conjugated bilirubin, hemoglobin, and lipid) on unconjugated bilirubin concentration measured by the UnaG method were also evaluated. Unconjugated bilirubin levels measured by the UnaG method were highly correlated with those determined by the bilirubin oxidase assay. Unconjugated bilirubin levels determined by bilirubin oxidase and UnaG assays were similar in serum samples containing conjugated bilirubin. The performance of the UnaG assay was unaffected by phototherapy and the presence of serum hemoglobin and lipid emulsion. These results demonstrate the clinical applicability of the UnaG method for direct measurement of unconjugated bilirubin levels in newborn serum.

Kernicterus or bilirubin-induced neurologic dysfunction is a brain disorder caused by bilirubin neurotoxicity during the neonatal period^1^. Not only does this disease show worldwide prevalence, its incidence is increasing in developed countries due to the higher survival rates of extremely preterm infants^2^,^3^. Although assessment of total bilirubin (TB) levels in serum/plasma is the current gold standard for identifying newborns at risk of kernicterus^4^,^5^, TB level is not the most accurate indicator, because TB includes both unconjugated bilirubin and conjugated bilirubin. Of these fractions, elevated serum level of unconjugated bilirubin in newborns is associated with increased risk of developing kernicterus^6^,^7^. Conventional methods have limited applicability since unconjugated bilirubin is insoluble in water at physiologic pH and temperature^8^,^9^; in current clinical settings, serum levels of unconjugated bilirubin are indirectly determined as the difference between total and conjugated bilirubin levels, which are measured with the diazo method, bilirubin oxidase method, or by spectrophotometry^10^. A major disadvantage of these conventional methods is to produce an inaccurate result in serum samples with turbidity. For example, the bilirubin oxidase method measures bilirubin levels by monitoring a change in absorbance at 450 nm, which is affected by turbidity due to hemoglobin or lipid emulsion^11^. High-performance liquid chromatography is the only method for directly measuring unconjugated bilirubin^12^, but is impractical for routine clinical use.

The recently cloned UnaG, fluorescent protein from eel muscle, specifically binds to the unconjugated but not the conjugated form of bilirubin with high affinity^13^. In this study, we used UnaG to directly measure unconjugated bilirubin levels in newborn serum or whole blood. Our findings demonstrate that the UnaG method is highly specific and sensitive and can be useful in a clinical setting.

## Results

### Reaction of UnaG and unconjugated bilirubin

**Figure 1:**
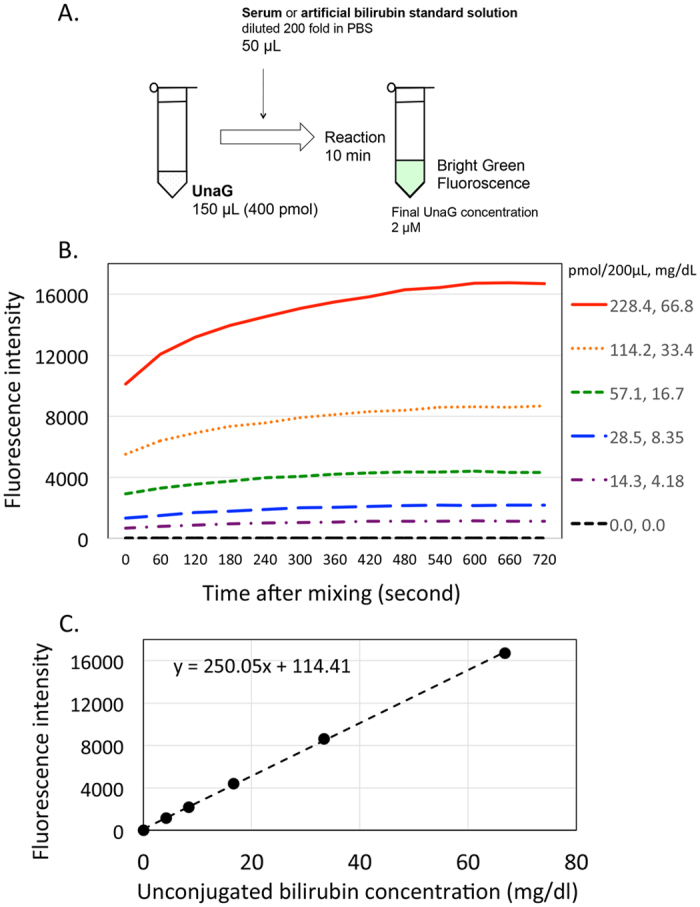
Reaction of UnaG with unconjugated bilirubin. (**A**) The UnaG assay procedure. (**B**) UnaG was reacted with artificial unconjugated bilirubin; green fluorescence intensity increased as a function of bilirubin concentration. (**C**) Calibration curve of fluorescence intensity as a function of unconjugated bilirubin concentration. PBS, phosphate-buffered saline; UnaG, fluorescent protein from eel muscle.

In this study, the fluorescence intensity of UnaG increased in the presence of artificial unconjugated bilirubin in a dose-dependent manner from 0 to ~70 mg/dl, with the intensity saturated around 10 min after mixing (Fig. 1B). The relationship between fluorescence intensity and unconjugated bilirubin concentration was linear in this range; an equation of the regression line was y = 250.05x + 114.41 (Fig. 1C). This range was consistent with those encountered in clinical settings.

### Precision of the UnaG method

Five serum samples with different concentrations of unconjugated bilirubin (A–E, 4.0, 11.5, 12.7, 15.0, and 16.7 mg/dl, respectively, by the bilirubin oxidase method) were analyzed to assess the precision of the UnaG method (Supplementary Table S1). All coefficients of variation (CV) values were <10%.

### Correlation between unconjugated bilirubin concentrations in serum obtained by bilirubin oxidase and UnaG methods

**Figure 2:**
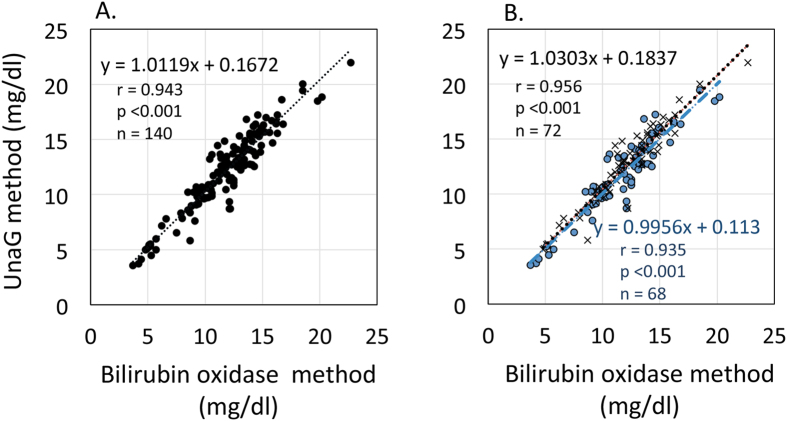
Correlation between serum unconjugated bilirubin concentrations measured by bilirubin oxidase and UnaG methods. (**A**) Serum samples without conjugated bilirubin (<1.0 mg/dl). (**B**) Effect of epitope tagging (UnaG-His-FLAG) on unconjugated bilirubin detection. Black crosses and blue circles represent data obtained using UnaG and UnaG-His-FLAG, respectively. UnaG, fluorescent protein from eel muscle.

A total of 140 serum samples obtained from 93 newborns with serum conjugated bilirubin concentrations <1.0 mg/dl were analyzed (median gestational age: 37 weeks, range: 27–41 weeks; median birth weight: 2760 g, range: 1014–3898 g; median age when blood sample was collected: 5 days, range: 1–35 days). The median serum conjugated bilirubin concentration determined by the bilirubin oxidase method was 0.2 mg/dl (range: 0.1–0.7 mg/dl). Unconjugated bilirubin concentrations measured by the UnaG and bilirubin oxidase methods were strongly correlated (y = 1.01x + 0.17, correlation coefficients (r) = 0.943, *P* < 0.001; Fig. 2A), and correlations were unaltered by using UnaG-His-FLAG (UnaG: y = 1.03x + 0.18, r = 0.956, *P* < 0.001, n = 72; UnaG-His-FLAG: y = 0.996x + 0.11, r = 0.935, *P* < 0.001, n = 68, *P* value between correlation coefficients = 0.25; Fig. 2B). Although we investigated whether the presence of the epitope tags have effect on the affinity of UnaG for unconjugated bilirubin, similar values were obtained using UnaG and UnaG-His-FLAG (Supplementary Table S2).

**Figure 3:**
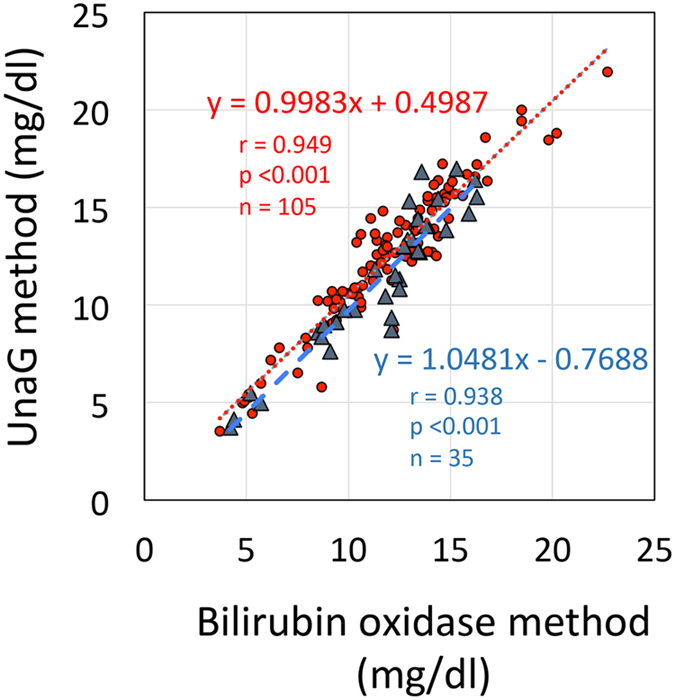
Correlation between unconjugated bilirubin concentrations measured by bilirubin oxidase and UnaG methods in newborns with/without phototherapy. Red circles and blue triangles represent data from newborns without and with phototherapy, respectively. UnaG, fluorescent protein from eel muscle.

There were no differences in the correlation between bilirubin oxidase and UnaG methods between newborns with and without phototherapy (PT) (without PT: y = 0.998x + 0.50, r = 0.949, *P* < 0.001, n = 105; with PT: y = 1.05x + 0.77, r = 0.938, *P* < 0.001, n = 35, *P* value between correlation coefficients = 0.62; Fig. 3).

### Influence of interfering factors in serum

Unconjugated bilirubin concentrations were similar across 14 serum samples with conjugated bilirubin concentration ≥1.0 mg/dl as determined by the UnaG and bilirubin oxidase methods (Table 1). There was no significant difference between values obtained with the bilirubin oxidase and UnaG methods (*P* = 0.31).

Next, we investigated whether substances such as hemoglobin and lipid emulsion in serum affect measurement of unconjugated bilirubin by the UnaG method. Five serum samples (a–e) with different concentrations of unconjugated bilirubin were analyzed. Unconjugated bilirubin concentrations as determined by the UnaG method were unchanged in the presence of various concentrations of hemolysate and lipid emulsion (Table 2). No correlation was found between hemoglobin or lipid emulsion concentrations and unconjugated bilirubin concentrations detected by the UnaG method (Samples a: r = −0.32, *P* = 0.54, b: r = −0.33, *P* = 0.53, c: r = 0.0032, *P* = 1.0, d: r = −0.62, *P* = 0.26, and e: r = −0.14, *P* = 0.82).

### Measurement of unconjugated bilirubin concentration in whole blood by the UnaG method

**Figure 4:**
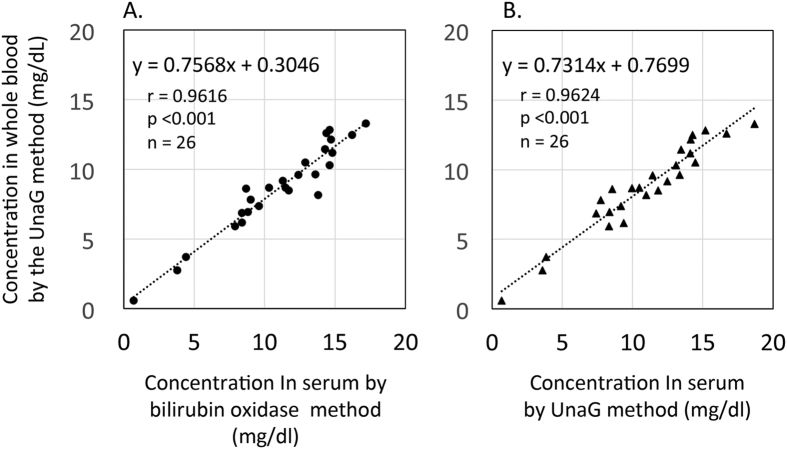
Unconjugated bilirubin concentrations in whole blood by the UnaG method. Linear regression analysis between unconjugated bilirubin concentrations (**A**) in serum by the bilirubin oxidase method and in whole blood by the UnaG method; and (**B**) in serum by the UnaG method and in whole blood by the UnaG method. UnaG, fluorescent protein from eel muscle.

A total of 26 whole blood samples from 18 newborns without PT were analyzed (median gestational age: 37 weeks, range: 27–40 weeks; median birth weight: 2432 g, range: 464–3558 g; median age when blood sample was collected: 5 days, range: 1–48 days). Median serum conjugated bilirubin concentration as determined by the bilirubin oxidase method was 0.2 mg/dl (range: 0.2–1.1); the median hematocrit value was 45% (range: 30–66%). Unconjugated bilirubin concentration in whole blood as determined by the UnaG method was strongly correlated with serum concentrations obtained by the bilirubin oxidase and UnaG methods (y = 0.76x + 0.30, r = 0.962 and y = 0.73x + 0.77, r = 0.962, respectively; *P* < 0.001; Fig. 4A,B); however, the slopes were not equal to 1 in either case.

## Discussion

This is the first large-scale clinical study demonstrating the applicability of eel-derived fluorescent protein for measurement of unconjugated bilirubin concentrations in newborn serum samples. The concentrations measured by this method showed high correlation with those obtained by the conventional bilirubin oxidase method, which has itself shown good correlation with those obtained by high-performance liquid chromatography, considered as the gold standard^12^,^14^,^15^. The performance of the UnaG method was unaffected by PT and the presence of serum hemoglobin and lipid emulsion. Moreover, only a small volume of whole blood or serum was required for the assay, which is advantageous since samples are collected from neonates. The precision of the method was confirmed by intra- and inter-day assays, which is an important factor for the clinical applicability of this method. These results indicate that the UnaG assay is robust and can be used for direct measurement of unconjugated bilirubin in newborns.

PT is a standard and effective treatment for severely jaundiced newborns^16^ and works by inducing the isomerization of *trans*-bilirubin into the water-soluble *cis* isomer, which is excreted from the body^17^. Detection of unconjugated bilirubin by the UnaG method was unaffected by PT. Importantly, UnaG bound the unconjugated but not the conjugated form of bilirubin^13^. There was no siginificant discrepancy between unconjugated bilirubin levels measured by the UnaG and bilirubin oxidase methods.

Hemolytic jaundice resulting from blood group incompatibility, glucose-6-phosphate dehydrogenase deficiency, or hereditary spherocytosis is a condition associated with a high risk of developing kernicterus^18^. In these cases, bilirubin levels in hemolysate must be measured. Parenteral alimentation with lipids is normally provided as nutrition to preterm infants or sickly newborns^19^. Although the bilirubin oxidase method is affected by the presence of serum hemoglobin and lipid emulsion^11^, we found in this study that hemolysate or serum containing emulsion did not affect unconjugated bilirubin measurement by the UnaG method. Additional studies are required to determine whether the various drugs used in clinical neonatal medicine interfere with UnaG-based detection.

The clinical applicability of the UnaG method depends on the ability to obtain UnaG in substantial quantities. His-tag DNA technology is often used to generate large amounts of specific proteins^20^. We thus generated UnaG-His-FLAG and tested its efficacy in the UnaG assay. The presence of the epitope tags had no effect on the affinity of UnaG for unconjugated bilirubin. These results demonstrate the feasibility of large-scale UnaG production. An automated measurement system for UnaG would also be useful for clinical application of this method.

We measured unconjugated bilirubin concentration in whole blood using the UnaG method with hematocrit value correction^13^^,^^21^ and found that the concentration was highly correlated with those measured in serum by the bilirubin oxidase and UnaG methods; however, the slopes of the linear regression lines were not equal to 1. This may be attributed to the fact that we used a standard curve generated using serum bilirubin solutions. To obtain corresponding values in serum and whole blood, appropriate standards and corresponding coefficient values must be used.

In conclusion, we established a highly specific and sensitive assay for detection of unconjugated bilirubin in serum samples from newborns using the fluorescent protein UnaG. The UnaG method yielded results that were at least comparable to those obtained by the conventional bilirubin oxidase method, in addition, this method is unaffected by the presence of serum hemoglobin and lipid emulsion.

## Methods

### Blood sample collection

Blood samples were obtained from newborns who were born between June in 2014 to December in 2015 for routine laboratory tests for a variety of reasons, such as respiratory disorder, neonatal jaundice, and conditions associated with premature birth. Residual blood samples after sampling for laboratory tests were immediately centrifuged at 3000 rpm for 10 min in the dark, and the serum was stored at −20 °C in the dark until it was used. The residual whole blood was stored at room temperature in the dark and used on the same day that the sample was collected. The study protocol was approved by the ethical committee of Kobe University Graduate School of Medicine (approval no. 1618). Informed consent was obtained from parents of newborns prior to collection of blood samples. The methods were carried out in accordance with the approved guidelines.

### Preparation of UnaG

The bilirubin-inducible fluorescent protein from Japanese eel muscle, UnaG was used in this study as we have previously shown^13^. UnaG-His-FLAG was generated by adding a polypeptide tag to the protein as previously reported^20^.

### UnaG method of unconjugated bilirubin detection

Detection in serum: A 200-μl reaction mixture containing 150 μl UnaG solution (400 pmol) and 50 μl diluted bilirubin solution or serum (for a final UnaG concentration of 2 μM) was prepared, with 5 μl artificial unconjugated bilirubin solution or newborn serum added to 995 μl of 0.1 M phosphate-buffered saline (PBS; 200-fold dilution) as we have previously shown (Fig. 1A)^13^. A microplate reader (SH-9000; Corona Electric Co., Hitachinaka, Japan) was used at 37 °C with fluorescence filters for excitation and emission wavelengths of 498 and 527 nm, respectively. Bilirubin concentrations ranging from 0.0 to 66.8 mg/dl were prepared using artificial bilirubin standard solutions including albumin (Arrows Co., Osaka, Japan) and were measured to generate a standard calibration curve (Fig. 1B,C). Serum samples were diluted 200 fold with PBS and fluorescence intensity was measured. Serum concentrations of unconjugated bilirubin were extrapolated from the standard curve.

Detection in whole blood: Artificial unconjugated bilirubin solution or newborn whole blood was diluted 100 fold in PBS. The fluorescence intensity was measured and a standard curve was generated using artificial unconjugated bilirubin solution as described above. Fluorescence intensity of diluted whole blood samples was measured and unconjugated bilirubin concentrations were determined from the standard calibration curve with a correction of (1 − hematocrit value). This correction method is generally used to covert to the serum concentration from the whole blood concentration^13^^,^^21^.

### Precision of the UnaG method

Intra-day precision was determined by analyzing six replicates of the serum sample over the course of 1 day; inter-day precision was estimated by analyzing the same sample on 6 difference days. Mean, standard deviation, and CV were calculated^22^.

### Bilirubin oxidase method of serum unconjugated bilirubin detection

Total and conjugated bilirubin concentrations in serum were measured using IatroLQ T-bil and IatroLQ D-bil kits (Unitika Co., Okazaki, Japan) as previously reported^14^,^15^. Serum unconjugated bilirubin concentration was calculated with the following formula (1 mg/dl = 17.1 μM): [unconjugated bilirubin] = [TB] – [conjugated bilirubin].

### Comparison between bilirubin oxidase and UnaG methods

Serum unconjugated bilirubin concentrations without conjugated bilirubin (<1.0 mg/dl by the bilirubin oxidase method) as determined by the UnaG and bilirubin oxidase methods were compared by linear regression analysis. The correlation between serum unconjugated bilirubin concentrations determined using UnaG and UnaG-His-FLAG was determined to verify whether these two forms of UnaG have different affinities for unconjugated bilirubin. Serum unconjugated bilirubin concentrations in the same serum samples were also determined using UnaG and UnaG-His-FLAG, with a comparison of the results. Unconjugated bilirubin concentrations in the serum of newborns treated with PT (blue light-emitting diodes, irradiance with around 30 μW/cm^2^/nm) or untreated were compared, since PT induces the photoisomerization of unconjugated bilirubin^17^,^23^.

### Influence of interfering factors in serum

Serum samples (with ≥1.0 mg/dl conjugated bilirubin by the bilirubin oxidase method) were obtained from newborns experiencing severe asphyxia with hepatic failure, congenital cytomegalovirus infection, or trisomy 18. Unconjugated bilirubin concentrations in these samples were measured by the UnaG or bilirubin oxidase methods. Interfering factors in the serum (hemoglobin and lipid) were evaluated to determine whether they affect unconjugated bilirubin detection by the UnaG method. Hemolysates were prepared as follows. Erythrocytes obtained from fresh blood of a volunteer adult were washed with saline and hemolysates with various concentrations of hemoglobin (0, 1, 2, 4, 8, and 16 g/dl) were prepared by adding appropriate volumes of distilled water, and were added to newborn serum samples to determine whether hemolysis can affect the measurement of UnaG fluorescence intensity. Similarly, serum samples with various concentrations of lipid emulsion (0, 0.02, 0.2, 2, and 20% intralipos; Otsuka Pharmaceutical Factory, Naruto, Japan) were used to assess whether these factors affect measurement of unconjugated bilirubin concentration.

### Analysis of unconjugated bilirubin concentrations in whole blood

Serum unconjugated bilirubin concentrations determined by the bilirubin oxidase and UnaG methods and the concentration in whole blood determined by the UnaG method were compared by linear regression analysis.

### Statistical analysis

Clinical data are presented as median (range) values. Linear regression analysis was performed and Pearson’s correlation coefficients were determined using Excel Statistics (Statcel 3; Social Survey Research Information Co., Tokyo, Japan). The Mann-Whitney U test implemented in Excel Statistics was used to compare two independent data sets. Differences were considered statistically significant for *P* < 0.05.

## Additional Information

**How to cite this article**: Iwatani, S. *et al.* Fluorescent protein-based detection of unconjugated bilirubin in newborn serum. *Sci. Rep.* **6**, 28489; doi: 10.1038/srep28489 (2016).

## Supplementary Material

Supplementary Information

## Figures and Tables

**Table 1:** Comparison of unconjugated bilirubin concentrations in serum containing conjugated bilirubin measured by the bilirubin oxidase and UnaG methods.

Sample	Bilirubin oxidase method (mg/dl)^†^			UnaG method (mg/dl)	Difference (mg/dl)*
	Total bilirubin	Conjugated bilirubin	Unconjugated bilirubin		
# 1	9.1	1.0	8.1	9.0	−0.9
# 2	14.7	1.2	13.5	15.0	−1.5
# 3	9.6	1.5	8.1	8.7	−0.6
# 4	9.1	2.1	7.0	7.1	−0.1
# 5	3.9	2.4	1.5	0.6	0.9
# 6	5.5	3.5	2.0	0.8	1.2
# 7	5.9	4.6	1.3	1.3	0.0
# 8	6.0	4.7	1.3	1.2	0.1
# 9	7.9	5.5	2.4	1.7	0.7
# 10	8.0	6.0	2.0	1.7	0.3
# 11	12.0	9.6	2.4	1.5	0.9
# 12	19.7	16.4	3.3	2.0	1.3
# 13	21.0	17.7	3.3	2.1	1.2
# 14	25.0	22.0	3.0	2.0	1.0
		Mean	4.2	3.9	0.3
		SD	3.46	4.17	0.84

SD, standard deviation; UnaG, fluorescent protein from eel muscle.

^†^Serum unconjugated bilirubin concentrations were determined by the bilirubin oxidase method with the following formula: [unconjugated bilirubin] = [total bilirubin] − [conjugated bilirubin] (1 mg/dl = 17.1 μM).

^*^Difference in values between bilirubin oxidase and UnaG methods.

**Table 2:** Effect of interfering factors present in serum on unconjugated bilirubin concentration measured by the UnaG method.

Hemolysis			
Sample	Hemoglobin (g/dl)		Unconjugated bilirubin (mg/dl)
a	16		11.4
	8		12.5
	4		11.3
	2		12.2
	1		12.3
	0		11.7
b	16		8.4
	8		9.1
	4		9.1
	2		8.8
	1		8.8
	0		8.5
c	16		4.7
	8		4.9
	4		5.3
	2		4.6
	1		5.0
	0		4.5
**Lipid**			
**Sample**		**Lipid emulsion (%)**	**Unconjugated bilirubin (mg/dl)**
d		20	15.5
		2	17.0
		0.2	16.3
		0.02	15.8
		0	16.5
e		20	8.9
		2	8.9
		0.2	8.7
		0.02	9.6
		0	8.8

Serum samples a–e contained 12.8, 9.1, 5.3, 16.9, and 7.8 mg/dl unconjugated bilirubin, respectively, as determined by the bilirubin oxidase method. Each concentration represents the final value after adding hemoglobin or lipid emulsion. UnaG, fluorescent protein from eel muscle.
