# Supplementary material for: Fluorescent protein-based detection of unconjugated bilirubin in newborn serum
Source: Sci Rep. 2016 Jun 21;6:28489. doi: 10.1038/srep28489 (PMC4914990; doi:10.1038/srep28489)
Supplement: Supplementary Information [file srep28489-s1.pdf]

Fluorescent protein-based detection of unconjugated bilirubin in newborn serum

Sota Iwatani[1], Hajime Nakamura[1], Daisuke Kurokawa[1], Keiji Yamana[1], Kosuke Nishida[1], Sachiyo Fukushima[1], Tsubasa Koda[1], Noriyuki Nishimura[1], Hisahide Nishio[2], Kazumoto Iijima[1], Atsushi Miyawaki[3], Ichiro Morioka[1*]

[1]Department of Pediatrics, Kobe University Graduate School of Medicine, Kobe 6500017, Japan
[2]Department of Epidemiology, Kobe University Graduate School of Medicine, Kobe 6500017, Japan
[3]Brain Science Institute, RIKEN, Wako 3510198, Japan

[*]Correspondence and requests for materials should be addressed to:
Ichiro Morioka, MD, PhD
Department of Pediatrics, Kobe University Graduate School of Medicine
7-5-2, Kusunoki-cho, Chuo-ku, Kobe 650-0017, Japan.
Phone: +81-78-382-6090, Fax: +81-78-382-6099, E-mail: ichim@med.kobe-u.ac.jp

Supplementary Table S1. Precision of the UnaG method

A. Intra-day assay

Sample	Bilirubin oxidase method (mg/dl)	UnaG method (mg/dl)						Median	Mean	SD	CV
		1	2	3	4	5	6				
#A	4.0	3.7	3.6	3.3	3.7	3.6	3.2	3.6	3.5	0.2	5.8
#B	11.5	11.1	10.7	10.7	10.6	10.7	10.7	10.7	10.7	0.2	1.5
#C	12.7	12.1	12.2	12.7	12.5	12.3	12.3	12.3	12.4	0.2	1.7
#D	15.0	15.2	14.8	15.1	14.8	14.3	14.1	14.8	14.7	0.4	3.0
#E	16.7	15.0	16.4	14.9	16.1	17.0	16.0	16.0	15.9	0.8	5.2

B. Inter-day assay

Sample	Bilirubin oxidase method (mg/dl)	UnaG method (mg/dl)						Median	Mean	SD	CV
		Day 1	Day 2	Day 3	Day 4	Day 5	Day 6				
#A	4.0	3.9	3.8	4.1	3.7	3.6	4.4	3.9	3.9	0.3	8.1
#B	11.5	13.5	10.8	12.4	11.1	11.7	11.8	11.7	11.9	1.0	8.3
#C	12.7	13.6	12.9	13.6	12.1	14.3	13.3	13.4	13.3	0.7	5.6
#D	15.0	14.7	15.2	14.1	15.3	15.1	14.5	14.9	14.8	0.5	3.2
#E	16.7	17.2	15.7	17.0	15.0	16.2	16.0	16.1	16.2	0.8	5.2

The measurements were performed independently 6 times. CV, coefficient of variation; SD, standard deviation; UnaG, fluorescent protein from eel muscle.

Supplementary Table S2. Measurement of unconjugated bilirubin in five selected serum samples by the UnaG method using UnaG or UnaG-His-FLAG

Sample	# I	# II	# III	# IV	# V
UnaG	3.4	6.6	8.5	12.2	13.8
UnaG-His-FLAG	3.3	6.4	8.5	12.3	13.7

UnaG, fluorescent protein from eel muscle.
